# Patterns of Raised Blood Pressure in Vietnam: Findings from the WHO STEPS Survey 2015

**DOI:** 10.1155/2019/1219783

**Published:** 2019-12-01

**Authors:** Van Minh Hoang, Quoc Bao Tran, Thi Hoang Lan Vu, Thi Kim Ngan Nguyen, Bao Giang Kim, Quynh Nga Pham, Tuan Lam Nguyen, Duc Truong Lai, Jun Nakagawa, Hai-Rim Shin, Warrick Junsuk Kim, Leanne Riley, Christina Wadhwani, Dinh Bac Truong, Dac Phu Tran

**Affiliations:** ^1^Hanoi University of Public Health (HUPH), Hanoi, Vietnam; ^2^Division of Non-Communicable Diseases, General Department of Preventive Medicine, Ministry of Health, Hanoi, Vietnam; ^3^Hanoi Medical University, Hanoi, Vietnam; ^4^World Health Organization Country Office, Hanoi, Vietnam; ^5^World Health Organization, Regional Office for the Western Pacific, Manila, Philippines; ^6^World Health Organization, HQ Office, Geneva, Switzerland; ^7^University of Basel, Basel, Switzerland

## Abstract

This study aims to describe the prevalence of raised blood pressure and the situation of management for raised blood pressure among the adult population in Vietnam. It also aims to examine the association between diversified socioeconomic and behavioral factors of raised blood pressure and awareness of raised blood pressure. Data were obtained from the STEPS survey conducted in Vietnam in 2015. Survey sample was nationally representative with a total of 3,856 people aged 18–69 years old. The study outcomes included raised blood pressure and awareness of and control of raised blood pressure. Multiple logistic regression was used to examine the association of socioeconomic and behavior risk factors with the outcome variables. The overall prevalence of raised blood pressure in Vietnam in 2015 was 18.9% (95% CI: 17.4%–20.6%). The prevalence of raised blood pressure was higher among men. Significantly correlated factors with raised blood pressure were age, sex, body mass index, and diabetes status. Levels of awareness of raised blood pressure were higher among the older age group and overweight people and lower among ethnic minority groups. Raised blood pressure in Vietnam is a serious problem due to its magnitude and the unacceptably high unawareness rate in the population. Public health actions dealing with the problems of raised blood pressure are urgent, while taking into account its relationship with sex and socioeconomic status. It is clear that the interventions should address all people in society, with a focus on disadvantaged groups which are the rural and ethnic minority peoples.

## 1. Introduction

High blood pressure (or raised blood pressure), known as a “silent killer,” is associated with approximately 9.4 million cases of death a year worldwide [1]. Traditionally, raised blood pressure has been estimated based on the definition of systolic blood pressure ≥140 mmHg and/or diastolic ≥90 mmHg or currently on medication for high blood pressure [2]. In 2013, the World Health Assembly adopted the global noncommunicable diseases (NCD) monitoring framework in which raised blood pressure is defined as systolic blood pressure ≥140 mmHg and/or diastolic ≥90 mmHg, regardless of whether the individual is on medication for high blood pressure. With this definition, the global prevalence of raised blood pressure in adults aged 18 years and above was around 22% in 2014 [3]. The advantage of the new definition is that each country will be able to reduce the prevalence of raised blood pressure if it can effectively control blood pressure in hypertensive patients. However, this indicator will not tell the size of the problem which includes those who are currently on medication. Therefore, in this paper, we will use the traditional definition of raised blood pressure which will include those who are currently on medication. The increase in prevalence of raised blood pressure is associated with population ageing and the rise in occurrence of behavioral risk factors, such as tobacco use, unhealthy diet, harmful use of alcohol, physical inactivity, overweight, and persistent stress [4]. Many people with high blood pressure in low-and middle-income countries are not aware of their hypertensive status, and lack access to treatment, and management services [4].

Vietnam has been undergoing health transition where populations are suffering from a “double burden” of communicable diseases and NCDs, including raised blood pressure [5, 6]. There were a few surveys aiming to access the level of raised blood pressure in Vietnam but they were only covering a small number of provinces, with different sampling methods and using different age ranges. For example, a cross-sectional survey with a sample from eight provinces and cities in Vietnam were conducted during 2002–2008 by the Vietnam National Heart Institute, covering the age range of 25 and above [7]. The World Health Organization (WHO) STEPwise approach to Surveillance (STEPS) of chronic disease risk factors in Vietnam 2009–2010 was taken among those aged 25–64, also from 8 provinces [8].

In 2015, for the first time, a WHO STEPS survey was conducted in a nationally representative sample which provided a good opportunity for Vietnam to establish a national prevalence and to explore factors related to raised blood pressure and its control.

Therefore, we aim to describe the prevalence of raised blood pressure and the situation of awareness and control of raised blood pressure among the adult population in Vietnam. We also aim to examine the association between diversified socioeconomic and behavioral factors of raised blood pressure and awareness of raised blood pressure.

## 2. Methods

### 2.1. Data Source

Data for this paper were obtained from the WHO STEPS survey conducted in Vietnam in 2015. This was a cross-sectional survey applying the standard methods and tools provided by the World Health Organization [9]. The survey was conducted in a sample from all 63 provinces/cities of Vietnam between June and October 2015. Survey participants were Vietnamese people aged 18–69 years old who were residing in Vietnam at the time conducting the survey. Exclusion criteria included (1) those who were not residing permanently in Vietnam; (2) patients who were admitted at a health facility at the time of the survey; and (3) people with mental health problems who cannot respond to the survey questions.

Sample size calculation was done using the sample calculator for STEPS provided by WHO [9]. A total of 3,856 people aged 18–69 years were selected for the survey. The two-stage random systematic sampling method was used. In the first stage, primary sampling units (PSUs) which included a cluster of households was identified. In the second stage, random selection of individual households within each cluster was done. In each household, one person between the ages of 18 and 69 was selected using the Kish Grid method. The sampling frame for the survey was developed by the General Statistics Office of Viet Nam (GSO) based on the master sampling frame used in the Population and Housing Census 2009 and updated with data of 2014. The first stage of the survey was conducted in the household using a questionnaire. The second (physical measurement) and third stages (biochemical tests) were conducted in a commune health station after the interview was conducted at the households.

### 2.2. Dependent Variables

Raised blood pressure was defined as an average (based on STEPS rule) systolic blood pressure (SBP) ≥140 mmHg and/or average diastolic blood pressure (DBP) ≥90 mmHg and/or self-reported current medication for high blood pressure in the previous two weeks. Digital automatic devices recommended by the WHO (BOSO device) were used for blood pressure measurements.

Management of raised blood pressure was measured by three indicators: awareness of high blood pressure (participants with raised blood pressure who were aware of their condition), treatment of high blood pressure (participants with raised blood pressure who reported current use of antihypertensive medications), and control of high blood pressure (participants who are currently on medication for raised blood pressure and have a blood pressure below 140/90 mmHg).

### 2.3. Independent Variables

Demographic variables were included, such as (1) sex (male and female); (2) age groups (i.e., 18–29, 30–49, and 50–69); (3) educational level (primary or less, secondary, and university/college); (4) occupation (senior officials and professionals, elementary occupations, and others); (5) residence (urban and rural); and (6) economic status of household, measured based on an asset-based wealth index lower income (two poorest quintiles), middle income (third and fourth quintiles), and higher income (fifth quintile) [10]. Behavioral risk factors for NCDs were also examined, including (1) smoking status (smoker and nonsmoker), (2) drinking status: drinker (consumed at least one standard drink of alcohol in the past 30 days) and nondrinker, and (3) overweight: people had body mass index (BMI) equal to or greater than 25, and non-overweight: BMI <25.

### 2.4. Data Analysis

Descriptive analysis was used to estimate prevalence, and multiple logistic regression was used to model the associations between outcomes variables and sociodemographic factors. Data were analyzed using Epi Info 3.54 and Stata 13 software. Because we used two-stage sampling, the sampling probability is calculated separately for each PSU and household. All the results presented reflect the survey design and survey weights. In order to account for sampling error, Taylor-linearized standard errors are used to estimate all 95% confidence intervals reported in Tables 1 and 2. The Taylor-linearized standard errors were calculated in Stata using the svy procedure. The reported 95% CI can be interpreted as the range in which 95% of proportions for a given variable would fall among all the possible samples drawn from the same population using the same design. For multiple regression model, the backward selection procedures were used to select a suitable set of independent variables, with the cutoff point to eliminate a variable being *p* > 0.2. For multiple regression model fitness, the estat gof goodness of fit was used.

### 2.5. Ethical Considerations

The protocol of the STEPS survey was approved by the Institutional Review Board of Hanoi University of Public Health. All human subjects in the study were asked for their written informed consent before data were collected, and all had the right to withdraw from the study at any time.

## 3. Results

Of the 3,856 selected participants, 3,758 responded to the survey (response rate of 97.4%) and participated in stage 1 of the survey. However, the number of study subjects who participated in the second and third stages of the survey was only 3,080 (response rate of 79.9%). The general characteristics of those study participants who participated in stages 2 and 3 are described in Table 3.

Table 1 presents the prevalence of hypertension by some sociodemographic factors. Data showed that the prevalence of hypertension increased significantly with age (5.4%, 17.3%, and 40.2% among people aged 18–29, 30–49, and 50–69, respectively). Male population had higher prevalence of hypertension compared to female population (23.1% vs. 14.9%). The prevalence of hypertension also varied as a function of three NCD risk factors, smoking, high BMI, and drinking. Specifically, among population with BMI less than 25, the prevalence of hypertension was 16.0% while among population with BMI greater than/equal to 25, this figure was 36.7%. About 22% people currently smoking and 21.6% people currently drinking were reported to have hypertension.

Table 2 provides a situational analysis about the level of awareness, treatment, and control of hypertension among people detected with hypertension in this survey. The prevalence of awareness was the percentage of people detected with hypertension in this survey who were aware of their condition; the prevalence of treated with medication was the percentage of people detected with hypertension in this survey who reported the current use of antihypertensive medications; the percentage of blood pressure controlled was the percentage of people detected with hypertension in this survey, who are currently on medication for raised blood pressure and have a blood pressure below 140/90 mmHg. There was a pattern of lower level of awareness, on medication and having blood pressure controlled among men compared to women (35.8%, 20.5%, and 7.5% vs. 47.1%, 31.4%, and 13.0%, respectively); among rural area compared with urban areas (35.4%, 22.2%, and 7.8% vs. 49.9%, 30.1%, and 13.5%, respectively); among smokers compared with nonsmokers (34.5%, 17.8%, and 5.8% vs. 42.8%, 27.8%, and 11.4%, respectively); among drinkers compared with nondrinkers (35.3%, 20.0%, and 6.4% vs. 45.3%, 29.6%, and 13.0%, respectively); and among low-income compared with higher income (32.7%, 19.7%, and 7.8% vs. 44.4%, 34.2%, and 15.0%, respectively).

Multivariate logistic regression models were applied to examine the association between multiple behavioral and socioeconomic factors with raised blood pressure and awareness of high blood pressure. For the model of raised blood pressure, all variables were used in the model and the goodness of fit test showed good fit for the model. For the awareness of raised blood pressure model, the income variable was omitted to satisfy the requirement for the good of fitness test (the inclusion of this variable cause the model to fail the goodness of fit test).

As shown in Table 4, age was a statistically significant correlate of raised blood pressure. The prevalence increased with age and was statistically different among people aged 30–49 (OR = 3.54, 95% CI: 2.19–5.71) and 50–69 years old (OR = 11.36, 95% CI: 7.06–18.26) as compared to the 18–29 group. Males were associated with higher raised blood pressure (OR = 2.35, 95% CI: 1.77–3.14) compared to females. Similarly, overweight/obesity and people with diabetes status were shown to be statistically significant predictors of raised blood pressure with OR = 2.80 (95% CI: 2.12–3.69) and OR = 2.13 (95% CI: 1.51–3.55), respectively. There was no statistically significant difference in prevalence of raised blood pressure by economic condition of household and smoking status.

As for awareness of raised blood pressure, Table 4 shows that older age (OR = 4.20, 95% CI: 1.27–13.81 (age 50–69 vs. age 18–29)), overweight (OR = 1.79, 95% CI: 1.16–2.75), vs. non-overweight were associated with higher awareness of raised blood pressure. On the other hand, being ethnic minority (OR = 0.54, 95% CI: 0.30–0.97) was associated with lower level of awareness of raised blood pressure.

## 4. Discussion

The prevalence and associated factors of raised blood pressure and raised blood pressure management are of interest for health staffs, planners, and policy makers in Vietnam as well as in other similar settings in the world. The STEPS survey, which was conducted in 2015 with a national representative sample, showed that the overall prevalence of raised blood pressure in adults aged 18 to 69 was 18.9%. This indicates that the condition already affects a sizeable proportion of the adult population in Vietnam. The prevalence estimated in this study could not be compared with the previous surveys in Vietnam due to differences in sampling methods, age groups, and geographical coverage.

The prevalence of raised blood pressure in the present study is lower than the prevalence of raised blood pressure in selected countries and regions such as Africa (30.8% in 2010) [11], China (from 20.5% to 28.7%) [12–16], Labanon (29.3%) [17], and Turkey (24.8%) [18]. Compared with the results from the systematic review and meta-analysis in 2015 comprising data on 45 low- and middle-income countries, which reported the pooled raised blood pressure prevalence of 37.8% (95% CI: 35.0–40.6) in upper middle-income countries and 23.1% (95% CI: 20.1–26.2) in low-income countries, the prevalence in Vietnam is also much lower [19]. In contrast, the prevalence of raised blood pressure in a Tanzania community-based on a cross-sectional study in 2013 was 8.0% [20], which is much lower than in our study.

The difference in raised blood pressure in men and women (23.09% vs. 14.86%) in Vietnam found in this study was consistent with the findings from previous Vietnamese studies [5, 7, 8, 21]. This pattern was also comparable to other studies carried out in both developed and developing countries [3, 12, 14–18, 22]. In contrast to other studies, a meta-analysis of 45 countries found that there was no significant sex-difference in raised blood pressure prevalence [19]. A key predictor of blood pressure in many populations is age, and the present study found similar results to other international and Vietnamese studies [5, 7, 8, 12–15, 19–23] confirming age to be positively correlated with raised blood pressure. Our results correspond to previous research which found that people who are overweight [7, 11–13, 17, 19, 20], are smokers [22–24], and have diabetes [12, 13, 20, 25] were shown to have a higher risk for raised blood pressure.

In terms of socioeconomic correlates of raised blood pressure, there was no statistically significant difference in prevalence of raised blood pressure by occupational status, ethnicity, and economic condition of household, residency, and drinking status but showed that raised blood pressure was found to be associated with education level. Previous studies show inconsistent results related to these factors [13, 19, 20, 22, 23, 26].

This study also revealed that the rates of awareness, treatment, and control of high blood pressure in the study populations were low (43.1%, 24.9%, and 9.7%, respectively). This fact was also reported by other studies conducted in Vietnam [5, 7, 8, 21], India in 2003 [27], Bangladesh in 2004 [28], Thailand in 2006 [29], and China [13, 15, 30]. These results underscore the urgent need for developing strategies and measures for improving awareness, prevention, and control of high blood pressure in Vietnam and other countries with similar problems.

This study's result is consistent with the findings that potential factors associated with awareness were age [14, 23] and overweight/obese [12, 15]. Other important predictors of awareness are living area and being ethnic minority consistent with other studies [7, 12, 15, 23, 26]. However, this study has not confirmed previous research that sex is also a factor associated with awareness of raised blood pressure [7, 12, 23].

In summary, our findings showed that raised blood pressure in Vietnam is a serious problem both because of its magnitude and the unacceptably high unawareness rate in the population. Given the findings from this study, public health actions dealing with the problems of raised blood pressure (such as health education, early detection and management of cases in primary care level, etc.) are urgent, while taking into account its relationship with age and socioeconomic status. It is clear that the interventions should address all people in society, with a focus on disadvantaged groups such as ethnic groups and rural population. This study was just a starting point. Deeper and more sophisticated analyses are needed to understand the situation in more detail. Nonetheless, the simple but valuable information emerging from this study will certainly help in planning and policy-making processes.

## Figures and Tables

**Table 1:** Prevalence of hypertension by sociodemographic factors and NCD risk factors, Vietnam 2015.

	Number (%)	Hypertensive	Prevalence	95% CI
Overall	3080	673	18.9	17.4–20.6
Sex				
Men	1320 (49.5)	365	23.1	20.4–26.0
Women	1760 (50.5)	308	14.9	13.1–16.8
Age group				
18–29	489 (32.4)	47	5.4	3.1–7.7
30–49	1500 (43.6)	196	7.3	14.9–19.7
50–69	1091 (24.0)	430	40.2	36.5–43.8
Residence				
Urban	1380 (34.9)	310	18.4	16.3–20.8
Rural	1700 (65.1)	362	19.3	17.2–21.5
Ethnicity				
Kinh	2514 (80.2)	578	19.8	18.0–21.7
Others	566 (19.8)	95	15.5	12.4–19.1
Education				
Primary school or less	1248 (37.8)	306	22.4	19.7–25.3
Secondary school	1330 (45.2)	277	18.2	15.7–20.9
University/college	495 (17.0)	89	13.6	10.6–17.3
Occupation				
Senior officials and professionals	260 (8.7)	39	14.8	10.3–20.7
Elementary occupations	1504 (48.5)	309	19.1	16.8–21.6
Others	1316 (42.8)	325	19.6	17.3–22.0
Income				
Lower income	1493 (46.4)	328	19.3	17.1–21.9
Middle income	1055 (35.5)	225	17.6	14.9–20.6
Higher income	532 (18.1)	120	20.6	16.9–24.7
BMI				
BMI < 25	2523 (84.4)	469	16.0	14.4–17.7
BMI ≥ 25	520 (15.6)	202	36.7	31.8–41.9
Smoking status				
Nonsmoker	2354 (74.5)	493	17.9	16.3–19.7
Smoker	720 (25.5)	179	22.0	18.6–25.9
Drinking status				
Nondrinker	1861 (55.9)	368	16.8	14.9–18.9
Drinker	1219 (44.1)	305	21.6	19.0–24.5

**Table 2:** Proportions of hypertensive people who were aware of their hypertensive status, being treated with medications, and had their high blood pressure controlled, Vietnam 2015.

	Number of people with raised blood pressure	Percentage aware of hypertensive status (95% CI)	Percentage treated with medications (95% CI)	Percentage having blood pressure controlled^1^ (95% CI)
Overall	673	43.1 (39.4–46.8)	24.9 (21.0–29.3)	9.7 (7.0–12.4)
Sex				
Men	365	35.8 (30.1–42.0)	20.5 (15.9–25.9)	7.5 (4.7–10.4)
Women	308	47.1 (40.7–53.6)	31.4 (25.7–37.8)	13.0 (8.3–17.6)
Age group				
18–29	47	18.3 (5.7–45.2)	12.8 (4.0–34.1)	8.9 (0.0–21.1)
30–49	196	33.8 (27.4–40.8)	15.3 (10.7–21.4)	3.8 (0.9–6.7)
50–69	430	49.4 (44.2–54.7)	34.4 (29.4–39.8)	14.4 (10.4–18.3)
Residence				
Urban	310	49.9 (42.2–57.5)	30.1 (24.2–36.7)	13.5 (9.6–18.7)
Rural	362	35.4 (30.5–40.8)	22.2 (17.3–28.1)	7.8 (5.0–11.9)
Ethnicity				
Kinh	578	43.7 (38.9–48.5)	26.3 (22.0–31.1)	10.6 (7.9–14.0)
Others	95	22.0 (13.9–33.1)	17.1 (9.4–29.0)	5.0 (1.7–14.1)
Education				
Primary school or less	306	39.6 (33.7–45.9)	26.6 (20.7–33.5)	10.4 (6.9–15.3)
Secondary school	277	40.9 (34.0–48.3)	23.5 (18.0–30.1)	9.9 (6.4–15.0)
University/college	89	41.2 (29.9–53.6)	23.8 (15.4–34.7)	6.6 (2.9–14.3)
Occupation				
Senior officials and professionals	39	46.4 (28.5–65.2)	28.6 (14.1–49.4)	8.3 (3.3–19.6)
Elementary occupations	309	33.6 (28.1–39.5)	18.4 (13.7–24.2)	7.8 (4.9–12.1)
Others	325	46.9 (40.3–53.6)	31.5 (25.7–37.9)	12.1 (8.6–16.6)
Income				
Lower income	328	32.7 (26.9–39.0)	19.7 (15.0–25.3)	7.8 (5.0–11.9)
Middle income	225	48.8 (40.6–57.1)	26.7 (21.0–33.4)	9.4 (6.3–13.9)
Higher income	120	44.4 (35.2–53.8)	34.2 (24.9–44.9)	15.0 (8.7–24.7)
BMI				
BMI < 25	469	35.6 (30.8–40.7)	18.7 (14.9–23.3)	8.9 (6.2–12.7)
BMI ≥ 25	202	51.2 (43.1–59.1)	38.7 (30.8–47.3)	11.5 (7.3–17.6)
Smoking status				
Nonsmoker	493	42.8 (37.8–47.9)	27.8 (23.3–32.9)	11.4 (8.4–15.3)
Smoker	179	34.5 (26.7–43.3)	17.8 (12.2–25.2)	5.8 (3.1–10.6)
Drinking status				
Nondrinker	368	45.3 (39.1–51.6)	29.6 (24.4–35.4)	13.0 (9.6–17.4)
Drinker	305	35.3 (29.0–42.2)	20.0 (15.2–25.9)	6.4 (3.8–10.6)

^1^Percentage among the hypertensive people.

**Table 3:** General characteristics of the study respondents, STEPS Vietnam 2015.

	Sample size and unweighted percentage, *n*	Weighted percentage
Gender		
Men	1320 (42.9)	49.5
Women	1760 (57.1)	50.5
Age group		
18–29	489 (15.9)	32.4
30–49	1500 (48.7)	43.6
50–69	1091 (35.4)	24.0
Residence		
Urban	1380 (44.8)	34.9
Rural	1700 (55.2)	65.1
Ethnicity		
Kinh	2514 (81.6)	80.2
Others	566 (18.4)	19.8
Education		
Primary school or less	1248 (40.5)	37.8
Secondary school	1330 (43.2)	45.2
University/college	495 (16.1)	17.0
Occupation		
Senior officials and professionals	260 (8.4)	8.7
Elementary occupations	1504 (48.8)	48.5
Others	1316 (42.7)	42.8
Income level		
Lower income	1493 (48.5)	46.4
Middle income	1055 (34.3)	35.5
Higher income	532 (17.3)	18.1
BMI		
BMI < 25	2523 (81.9)	84.4
BMI ≥ 25	520 (16.9)	15.6
Smoking status		
Nonsmoker	2354 (76.4)	74.5
Smoker	720 (23.4)	25.5
Drinking status		
Nondrinker	1861 (60.4)	55.9
Drinker	1219 (39.6)	44.1
Diabetes status		
Diabetes	155 (5.0)	4.1
Nondiabetes	2925 (95.0)	95.9
Total	3080 (100)	

**Table 4:** Multiple regression analyses: association between sociodemographic factors and hypertension and awareness of hypertension status in Vietnam, 2015.

	Having hypertension, OR (95% CI)	Aware of hypertension status, OR (95% CI)
Sex (women as reference)		
Men	2.35 (1.77–3.14)^*∗∗∗*^	
Age group (18–29 as reference)		
30–49	3.54 (2.19–5.71)^*∗∗∗*^	2.38 (0.70–8.13)
50–69	11.36 (7.06–18.26)^*∗∗∗*^	4.20 (1.27–13.81)^*∗*^
Residence (urban as reference)		
Rural		0.68 (0.44–1.05)
Ethnicity (Kinh as reference)		
Other minority groups		0.54 (0.30–0.97)^*∗*^
Income group using wealth index (lower income as reference)		
Middle-income	1.32 (0.83–2.09)	1.56 (0.97–2.50)
Higher-income	1.36 (0.86–2.15)	1.19 (0.70–2.03)
BMI (BMI less 25 as reference)		
Yes	2.80 (2.12–3.69)^*∗∗∗*^	1.79 (1.16–2.75)^*∗*^
Smoking (nonsmoker as reference)		
Yes	1.32 (0.95–1.82)	
Drinking (nondrinker as reference)		
Yes		0.71 (0.47–1.07)
Diabetes status (nondiabetes as reference)		
Yes	2.13 (1.51–3.55)^*∗∗∗*^	

^*∗*^ *p* < 0.05, ^*∗∗*^*p* < 0.01, ^*∗∗∗*^*p* < 0.001 (compared with the reference in each category).

## Data Availability

The data used to support the findings of this study are available from the corresponding author upon request.
